# Isolated recessive nail dysplasia caused by *FZD6* mutations: report of three families and review of the literature

**DOI:** 10.1111/ced.12934

**Published:** 2016-10-27

**Authors:** C. Kasparis, D. Reid, N. J. Wilson, V. Okur, C. Cole, C. D. Hansen, K. Bosse, R. C. Betz, M. Khan, F. J. D. Smith

**Affiliations:** ^1^ Dermatology DepartmentWalsall Healthcare NHS TrustWalsallUK; ^2^ Centre for Dermatology and Genetic MedicineDivision of Biological Chemistry and Drug DiscoverySchool of Life SciencesUniversity of DundeeDundeeUK; ^3^ Department of Medical GeneticsHaydarpasa Numune HospitalIstanbulTurkey; ^4^ Department of PediatricsColumbia University Medical CenterNew YorkNYUSA; ^5^ Division of Computational BiologySchool of Life SciencesUniversity of DundeeDundeeUK; ^6^ Department of DermatologyUniversity of UtahSalt Lake CityUTUSA; ^7^ Institute of Human GeneticsUniversity of BonnBonnGermany; ^8^ Institute of Medical Genetics and Applied Genomics and Department of Obstetrics and GynecologyUniversity Hospital of TübingenTübingenGermany; ^9^ Pachyonychia Congenita ProjectSalt Lake CityUTUSA

## Abstract

Congenital abnormalities of the nail are rare conditions that are most frequently associated with congenital ectodermal syndromes involving several of the epidermal appendages including the skin, teeth, hair and nails. Isolated recessive nail dysplasia (IRND) is much rarer but has recently been recognized as a condition resulting in 20‐nail dystrophy in the absence of other cutaneous or extracutaneous findings. A few case reports have identified mutations in the *Frizzled 6* (*FZD6*) gene in families presenting with abnormal nails consistent with IRND. These reports have highlighted the role of Wnt–FZD signalling in the process of nail formation. We report three families presenting with features of IRND, in whom we identified mutations in *FZD6*, including one previously unreported mutation.

Congenital abnormalities of the nail are rare disorders, often associated with congenital ectodermal syndromes involving several of the epidermal appendages including the skin, teeth, hair and nails. This is the case for pachyonychia congenita (PC), a disorder of keratinization and blistering involving the nails and skin. Despite its rarity, PC is perhaps one of the better understood, researched and reported congenital nail syndromes, whereas isolated recessive nail dysplasia (IRND) is much rarer and less well understood.^1^

The molecular pathways involved in the signalling for nail development are multiple and complex, thus mutations of genes involved in this process ultimately result in nail malformation.^2^ The *Frizzled* (*FZD*) gene family encodes FZD receptors, which act as receptors for the Wnt signalling proteins. The role of the Wnt–FZD pathway is increasingly recognized in nail formation. Recently, mutations in the *FZD6* gene have been identified as a cause of IRND [OMIM #614157; (NDNC)‐10] in several families.^3^, ^4^, ^5^, ^6^ We report three families in which individuals presented with a similar pattern of nail deformities, and were investigated for mutations of the *FZD6* gene.

## Report

All genetic studies were carried out after appropriate ethics approval, and the studies complied with the Declaration of Helsinki Principles. All participants (or their parent/guardians as appropriate) provided written informed consent.

**Figure 1:**
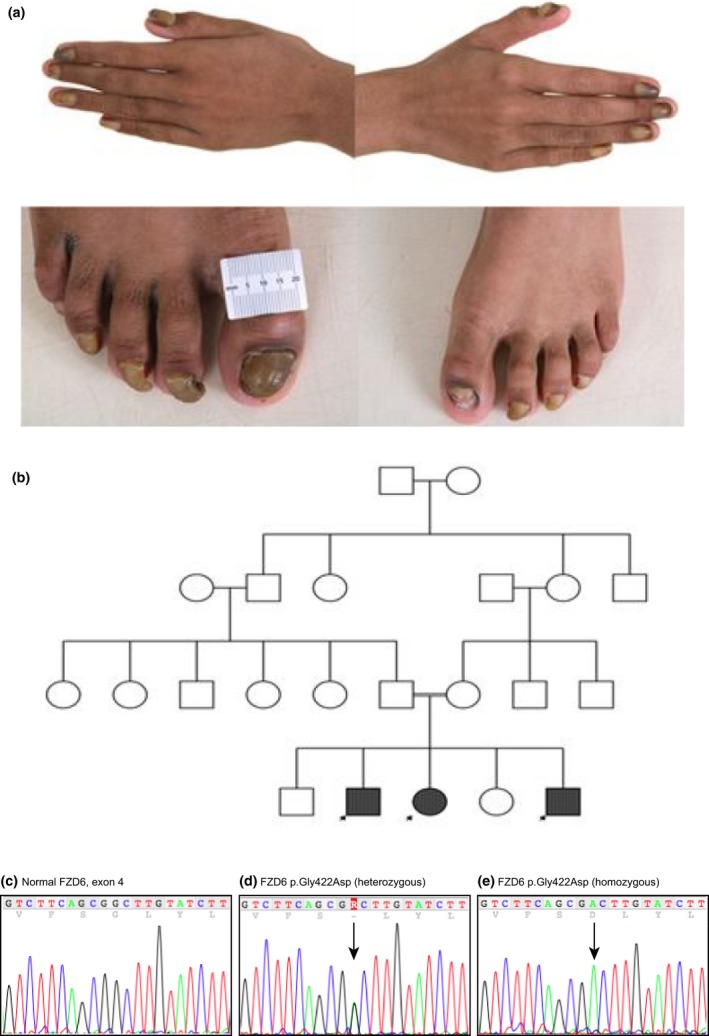
Family 1. (a) Twenty‐nail dystrophy in three siblings with marked subungual hyperkeratosis and onychogryphosis; the remaining two siblings in the family had normal nails. (b) The inheritance in this family follows an autosomal recessive pattern. (c–e) Sequence analysis: (c) a normal FZD6 sequence covering nucleotides c.1255‐1275; (d) equivalent region from the unaffected mother showing the heterozygous mutation c.1265G>A, leading to the missense mutation p.Gly422Asp; (e) equivalent region from one of the affected children, showing the same mutation in the homozygous state.

Family 1 was a consanguineous Pakistani family; three of the five siblings had abnormalities of their nails, inherited in an autosomal recessive pattern (Fig. 1). The nail problem was noticed in all affected siblings at 6–8 weeks of age. In the 3 affected siblings, all 20 nails were similarly affected, but there was a variable degree of nail dystrophy, manifesting primarily as subungual hyperkeratosis and onycholysis. The hyperkeratosis resulted in onychogryphosis and a claw appearance of several nails, together with yellow onychauxis of the plates. Nail growth was slow with occasional painful lifting and dropping off. The results of skin and systemic examinations were otherwise unremarkable. Both of the parents and the other two siblings had normal nails. Genomic DNA was obtained from blood samples, and amplified and sequenced for the *FZD6* gene (accession number NM_003506) as described by Wilson *et al*.^6^ A homozygous missense mutation p.Gly422Asp; c.1265G>A was identified in the *FZD6* gene in all three affected siblings (Fig. 1). The unaffected parents were found to be heterozygous carriers for this mutation, and one unaffected sibling had the wild‐type gene (the other unaffected sibling was not tested as no sample was obtained). This mutation has been previously reported as the cause of IRND in another family, also of Pakistani origin.^4^

**Figure 2:**
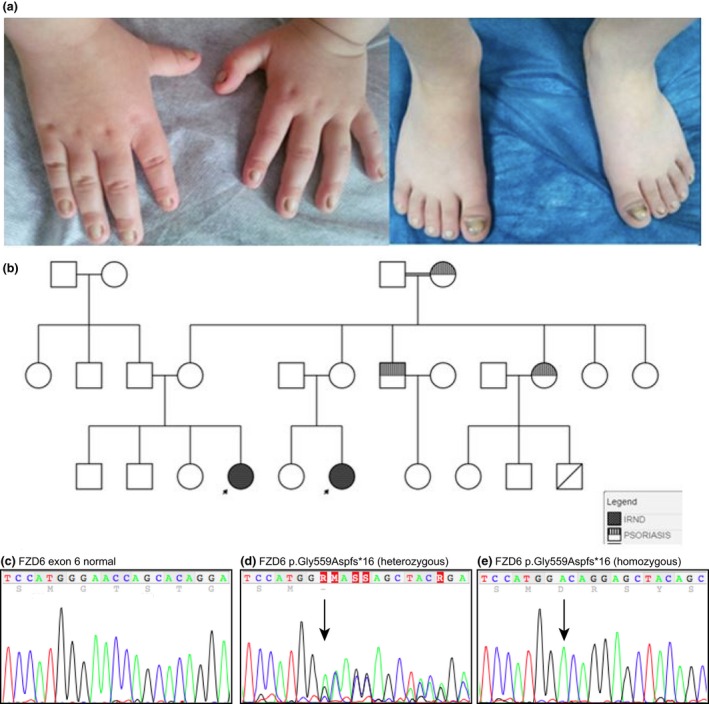
Family 2. (a) The two girls from Family 2 presented with a similar pattern of nail deformities; (b) inheritance for isolated recessive nail dysplasia in this family follows an autosomal recessive pattern (black). An incidental family history of psoriasis (shaded) was also noted. (c–e) Sequence analysis: (c) normal FZD6 sequence showing nucleotides c.1669‐1689; (d) equivalent region from the unaffected mother showing the heterozygous mutation c.1676_1683delGAACCAGC, leading to the frameshift mutation p.Gly559Aspfs*16; (e) equivalent region from one of the affected children, showing the same mutation in the homozygous state. This mutation was also identified in Family 3.

Family 2 was from a small village in Turkey and there was first‐degree consanguinity between the maternal grandparents (Fig. 2). The probands were two young girls who were cousins, and both presented with isolated nail dystrophy. Patient 1 developed overgrowth and abnormal conformation of all nails when she was 3 months old. There was a family history of psoriasis on her maternal side. Patient 2 presented at 3 years of age with similar features. Using direct DNA sequencing, both affected children were found to be homozygous for an 8 bp deletion mutation, p.Gly559Aspfs*16; c.1676_1683delGAACCAGC, in *FZD6* (Fig. 2). The mutation causes a frameshift and creates a premature stop codon at position 16 of the new reading frame. The unaffected parents of each child were heterozygous carriers for the mutation. This mutation has not been previously reported; it was not found in the dbSNP database, the 1000 Genomes Project or the NHLBI Exome Sequencing Project (http://evs.gs.washington.edu/EVS/). The *in silico* prediction tool, Mutation Taster, predicted this to be a disease‐causing variant, and that it will lead to a truncated protein or to protein loss via nonsense‐mediated decay.

**Figure 3:**
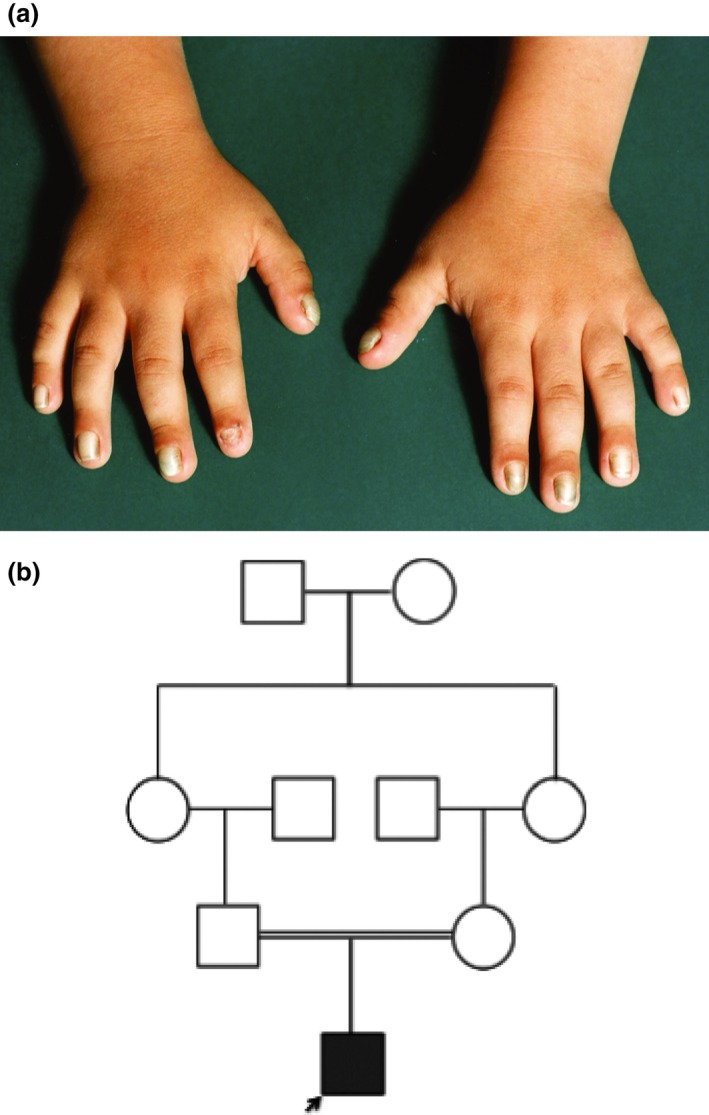
Family 3. (a) The affected boy had similar nail changes to those of the affected children in the other two families. (b) The pedigree for family 3 showing autosomal recessive inheritance of the mutation c.1676_1683delGAACCAGC.

Family 3 was a Turkish family living in Germany. The proband was a 3‐year‐old boy born to consanguineous unaffected parents. He was born with thickening of and dark edges to his nails (Fig. 3). By the age of 3 years there was marked elevation of the nail plate and he had already lost several nails. This family was screened by whole exome sequencing (Genomic Sequencing Unit, Dundee, UK), and the affected child was found to be homozygous for a deletion mutation p.Gly559Aspfs*16 in *FZD6*, the same mutation as that identified in Family 2 (Fig. 2). The mutation was confirmed by Sanger sequencing, and both parents were found to be heterozygous carriers.

Nail development begins around week 9 of the embryonic period, starting at the dorsal aspect of the distal end of the digits with mesenchymal condensation, which is shortly followed by the development of the transverse nailfold.^7^ Beneath the transverse nailfold the matrix primordium, containing proliferating keratinocytes, forms. This process initiates the development of the nail bed with the expression of epithelial keratins, while the keratinocytes eventually undergo apoptosis, resulting in the keratin cytoskeleton of the nail plate. The nail plate is known to originate in the apical matrix of the nail along with the keratogenous zone of the ventral matrix.^8^ The embryonic development of the ectodermal appendages, including the nails, relies on the Wnt–FZD signalling pathway.^4^ FZD6 is expressed in the keratogenous zone of the ventral matrix, where there is staggered expression of various keratins.^3^ It has therefore been postulated that *FZD6* mutations could lead to disorganization of the staggered expression of keratins, resulting in nail dysplasia.

The action of FZD6 at the molecular level in claw development in mice was investigated by Cui *et al*.,^9^ and their findings suggested a regulatory role for FZD6‐mediated Wnt signalling in the differentiation process of claw/nail formation. Recently, mutations in the *FZD6* gene have been identified as a key culprit in the development of IRND in several families.^3^, ^4^, ^5^, ^6^ In 2011, Frojmark *et al*.^5^ reported two consanguineous Pakistani families with some members affected by isolated nail dysplasia. The authors confirmed that homozygous *FZD6* mutations (p.Glu584* and p.Arg511Cys) result in nail deformities through reduced or aberrant FZD6 and nonfunctioning FZD‐Wnt pathways. A homozygous nonsense *FZD6* mutation (p.Glu584*) was also found in all affected members of two further families of Pakistani origin with IRND.^3^ The authors concluded that absence of nails is due to loss of activation of the Wnt/β‐catenin signalling cascade whereas nail overgrowth is caused by loss of inhibition of the Wnt/β‐catenin signalling cascade. Four additional unrelated families presenting with 20‐nail dystrophy caused by *FZD6* mutations have been described (homozygous p.Gly422Asp, homozygous p.Arg509* and compound heterozygous p.Arg96Cys/Glu438Lys), confirming the findings of previous studies.^4^, ^6^ To date, five different mutations in *FZD6* have been reported in eight families.

In this paper we report 3 further families with mutations in *FZD6* causing IRND. The initial clinical impression in all three families was PC. However, this was later rejected as all other ectodermal tissues showed no abnormalities. The recessive pattern of inheritance was also not consistent with PC. It became evident that the clinical findings were more in keeping with IRND, and genetic analysis was carried out. DNA sequence analysis identified one previously unreported mutation and one known mutation in *FZD6*. Families 2 and 3 are both of Turkish origin, although not knowingly related. The presenting clinical features of all affected individuals appear consistently similar to those of previous reports, with subungual hyperkeratosis, yellow onychauxis and a claw appearance of all 20 nails without any other cutaneous or appendageal signs.^3^, ^4^, ^5^, ^6^, ^10^ The pattern of autosomal recessive inheritance in all three families is in keeping with published case reports of IRND.

Our results enhance the existing but limited knowledge regarding the role of FZD6 mutations and the Wnt pathway in the pathogenesis of IRND. Dermatologists need to be aware of this entity, and be able to differentiate it from PC and other hereditary nail disorders. Genetic analysis of *FZD6* should be considered in all cases of isolated nail dysplasia. This will enable accurate genetic counselling of the family and avoidance of unnecessary treatments that have been used for PC. Further studies into the role of the Wnt signalling pathway are needed to provide more clarity of the pathophysiological picture of congenital nail deformities.

**Learning points**

- Anomalies in Wnt–FZD signalling pathways during early embryonic life result in nail dysplasia.
- IRND has been described recently in humans as a rare form of inherited nail dysplasia.
- Dystrophy of all 20 nails in the absence of cutaneous or appendageal features is a clue to the diagnosis.
- Six mutations in *FZD6* have so far been reported (including the one reported in this paper).
- Genetic analysis of *FZD6* should be considered in cases of sporadic or known recessive cases of isolated nail dysplasia.
